# Prognostic Value of Inflammatory and Tumour Markers in Small-Duct Subtype Intrahepatic Cholangiocarcinoma after Curative-Intent Resection

**DOI:** 10.1155/2021/6616062

**Published:** 2021-03-26

**Authors:** Bingqi Ma, Huijuan Meng, An Shen, Yuwen Ma, Dianpeng Zhao, Guiling Liu, Shujuan Zheng, Ye Tian, Wei Zhang, Qiang Li, Shiping Li

**Affiliations:** ^1^Department of Hepatopancreatobiliary Surgery, Affiliated Hospital of Weifang Medical University, Weifang 261031, China; ^2^Department of Dermatology, Affiliated Hospital of Weifang Medical University, Weifang 261031, China; ^3^Tianjin Medical University Cancer Institute and Hospital, National Clinical Research Center for Cancer, Key Laboratory of Cancer Prevention and Therapy, Tianjin, Tianjin's Clinical Research Center for Cancer, Tianjin 300060, China; ^4^Department of Hepatobiliary Surgery, Tianjin Medical University Cancer Institute and Hospital, National Clinical Research Center for Cancer, Key Laboratory of Cancer Prevention and Therapy, Tianjin, Tianjin's Clinical Research Center for Cancer, Tianjin 300060, China

## Abstract

Intrahepatic cholangiocarcinoma (ICC) is characterised by heterogeneity, and it can be subdivided into small-duct and large-duct types. Inflammatory and tumour markers could effectively predict prognosis in many cancers, but no similar studies have been conducted in the histological subtypes of ICC. A total of 102 and 72 patients with ICC undergoing curative-intent resection were retrospectively subclassified into large-duct and small-duct types by chemical staining, respectively. The prognostic value of inflammatory and tumour markers was studied for the first time in histological subtypes of ICC by using a Cox regression model. A novel predictor named prognostic inflammatory index (PII) was proposed and defined as neutrophil × monocyte/lymphocyte count (10^9^/L). Survival analysis showed that PII, neutrophil-to-lymphocyte ratio (NLR), lymphocyte-to-monocyte ratio (LMR), carcinoembryonic antigen (CEA), carbohydrate antigen 19-9 (CA19-9), CA242, and ferritin were all predictors of DFS and OS in patients with ICC (*P* < 0.040). Subgroup analysis showed that PII, CA19-9, and ferritin were risk predictors of disease-free survival (DFS) and overall survival (OS) in small-duct type ICC (*P* < 0.015). In addition, in small-duct type ICC, NLR and LMR were correlated with OS (*P* < 0.025), whilst CEA and CA242 were correlated with DFS (*P* ≤ 0.010). In conclusion, PII is a convenient and efficient inflammatory predictor of DFS and OS in ICCs and their small-duct type. NLR and LMR, rather than platelet-to-lymphocyte ratio, were correlated with OS in small-duct type ICC. In addition, ferritin may be a supplement to CA19-9 in stratifying the survival outcome of patients with small-duct type ICC.

## 1. Introduction

Intrahepatic cholangiocarcinoma (ICC) is one of the most frequent primary hepatic malignant tumours behind hepatocellular carcinoma, with an increasing incidence in the last three decades [1]. Surgical resection remains the main method of potentially curative treatment for patients with ICC. However, the 5-year overall survival (OS) rate of patients with ICC after curative-intent resection was 18%–36% [2, 3]. Meanwhile, the outcomes of different patients with ICC after surgery vary considerably. Although the TNM stage is a recognized predictor for patients with ICC, it is difficult to determine accurately before surgery. An efficient and simple biomarker for predicting postoperative survival in ICC is still lacking.

ICC is characterised by heterogeneity, and it can be subdivided into small-duct and large-duct subtypes on the basis of the level of the displayed bile duct, which has been widely accepted [4–6]. This classification not only has histological significance but also endows ICC subtypes with specific molecular and clinical characteristics [4]. For example, the large-duct type ICC showed a higher frequency of periductal-infiltrating growth pattern and perineural infiltration and higher pT stages than small-duct type ICC. Low-grade intraepithelial neoplasia in adjacent biliary ducts was exclusively seen in small-duct type ICC [5].

Some studies have shown that inflammation-based factors have a prognostic value in postoperative survival in patients with solid tumours, such as pancreatic cancer [7], hepatocellular carcinoma [8], and ICC [9]. These prognostic indices, including neutrophil-to-lymphocyte ratio (NLR), lymphocyte-to-monocyte ratio (LMR), platelet-to-lymphocyte ratio (PLR), prognostic scoring, Glasgow prognostic score (GPS), preoperative systemic inflammation score, systemic immune-inflammation index, and prognostic index, are based on C-reaction protein, blood routine examination, and albumin [9–13]. However, the prognostic ability of these biomarkers is not completely consistent in ICC. For example, Chen et al. [14] found that evaluated PLR was a risk factor for OS and disease-free survival (DFS) in patients with ICC, whilst Ohira et al. [15] showed that PLR was not correlated with postoperative survival.

Tumour markers are common means of aiding diagnosis, evaluating prognosis, and supervising recurrence. Carbohydrate antigen 19-9 (CA19-9) is the most valuable tumour marker in the assessment of ICC, although its increase may be caused by obstructive jaundice or cholangitis [16–21]. Carcinoembryonic antigen (CEA), a well-known biomarker in gastrointestinal cancers, has also received increased attention as a potentially effective biomarker for ICC [17–22]. CEA is independent of serum bilirubin levels, and it may have a predictive value for surgical resection rates. Two studies reported that ferritin may serve as a risk factor for postoperative survival of patients with ICC [23, 24].

Here, whether common inflammatory factors and tumour markers are associated with the prognosis and clinical features in small-duct and large-duct subtypes of ICC was investigated for the first time. Given the clear difference between large-duct and small-duct type ICC, subgroup analysis could obtain more accurate results than whole-group analysis. This study determined which biomarkers were predictors of postoperative recurrence and survival in small-duct type ICC and the difference in the predictive value of the same biomarker in different subtypes of ICC.

## 2. Methods

### 2.1. Patients

From January 2012 to December 2017, 102 consecutive patients with ICC who received potentially curative hepatectomy at Tianjin Medical University Cancer Institute and Hospital were retrospectively reviewed and analysed as a training cohort in this study. Meanwhile, a validation cohort was selected from 72 consecutive patients undergoing curative-intent resection for ICC at Affiliated Hospital of Weifang Medical University from January 2010 to June 2018. Patients with combined hepatocellular carcinoma–cholangiocarcinoma and those who underwent preoperative treatments, such as radiofrequency ablation or adjuvant chemotherapy, were excluded. Two groups of patients were reviewed to verify the ICC diagnosis and restage on the basis of the eighth edition TNM staging system. Demographic and baseline clinicopathological data, including preoperative blood routine examination, liver function, and tumour markers, were carefully collected and analysed. And preoperative blood samples were those collected after admission without any treatment (2–5 days before surgery). All patients were regularly followed up every 3 months in the first 2 years and 6 months thereafter. DFS was defined as the period between the date of surgery and the date of first recurrence or last follow-up, whereas OS was calculated from the date of first diagnosis of ICC to the date of death or last follow-up.

Tumour samples and pathological slices of 174 patients in the two cohorts were collected from the pathological tissue bank of the corresponding hospital.

The Medical Ethics Committees of Tianjin Medical University Cancer Institute and Hospital (Approval No.: bc2019065) and Affiliated Hospital of Weifang Medical University (Approval No.: 2020-02) approved this study. Informed consents were obtained from all patients or their legal guardian.

### 2.2. Histological Classification of ICC

In accordance with histological characteristics, S100P expression, and Alcian blue score, the patients with ICC were subdivided into small-duct type and large-duct type, as previously described [6]. Small-duct type is composed of cuboidal to low columnar tumour cells arranged in acinar or small-sized tubular pattern, and it usually shows scant S100P expression and mucin production. Large-duct type presents as a large-sized tubular or glandular component composed of tall columnar tumour cells, and it is usually characterised by abundant S100P expression and mucin production [4–6].

Finally, 81 (79.4%) and 21 (20.6%) cases were identified as small-duct type and large-duct type of ICC in the training cohort, respectively, whilst the validation cohort included 53 (73.6%) and 19 (26.4%) patients with small-duct and large-duct type ICC, respectively.

### 2.3. Statistical Analysis

Continuous variables were presented as medians or means and evaluated using Mann–Whitney *U* test or *t* test, as appropriate. Categorical variables, described as totals and frequencies, were compared using *χ*^2^ test or Fisher Exact test, as appropriate. Survival analysis (DFS and OS) was conducted using Kaplan–Meier and log-rank methods, whilst multivariate analysis was performed using a Cox regression model. The results of survival analysis were presented as hazard ratios (HRs) with 95% confidence intervals (CIs). Blood sample results at 2 to 5 days before surgery, 1 day after surgery, and 1 month after surgery were divided into two groups with the median as the cut-off value, respectively, and survival analysis was conducted to determine the predictive value of these indicators. The optimal cut-off values of preoperative inflammatory factors and tumour markers were analysed using the receiver operating characteristic (ROC) curve on the basis of a 3-year OS. A two-tailed *P* value < 0.05 was considered statistically different. SPSS 24 (Chicago, USA) was used for statistical analysis.

## 3. Results

### 3.1. Prognostic Value of Inflammatory Factors and Tumour Markers for ICC in the Training Cohort

The demographic and baseline clinicopathological data of patients with ICC in the training cohort are summarised in Table S1. A total of 102 patients (55.9% male and 44.1% female) were enrolled in this study, and their median age was 58 years. Only 55 cases (53.9%) presented lymphadenectomy. The median time of follow-up was 25.1 months (from 4.9 months to 100.0 months); 65 patients experienced tumour recurrence and 41 died at the end of follow-up. Survival analysis showed that the 1- and 3-year DFS rates were 60.0% and 30.4%, respectively, whilst the OS rates were 85.2% and 59.4%, respectively.

The optimal cut-off values of inflammatory factors (platelet, neutrophil, lymphocyte, monocyte, NLR, LMR, and PLR) and tumour markers (CEA, CA19-9, ferritin, and CA242) in this study were determined using the ROC curve on the basis of 3-year OS. In accordance with each of the aforementioned factors, all patients with ICC were classified into two subgroups for survival analysis.

Blood samples results collected 2 to 5 days preoperatively, 1 day postoperatively, and 1 month postoperatively were analysed for survival analysis. Statistical analysis found that only preoperative blood sample indexes were associated with the prognosis of ICC patients (data not shown). Therefore, both inflammatory factors and tumour markers in this study refer to the results of blood samples without any treatment after admission.

In the aspect of inflammatory factors, survival analysis exhibited that low lymphocyte and high monocyte were correlated with a trend toward inferior OS (*P* = 0.068) and DFS (*P* = 0.064) in the training set, respectively. Neutrophil, lymphocyte, monocyte, NLR, and LMR were all predictors of DFS and OS in patients with ICC (*P* ≤ 0.041). However, platelet and PLR were not related to DFS and OS in univariate analysis (*P* ≥ 0.065) (Table 1, Figure S1).

**Figure 1:**
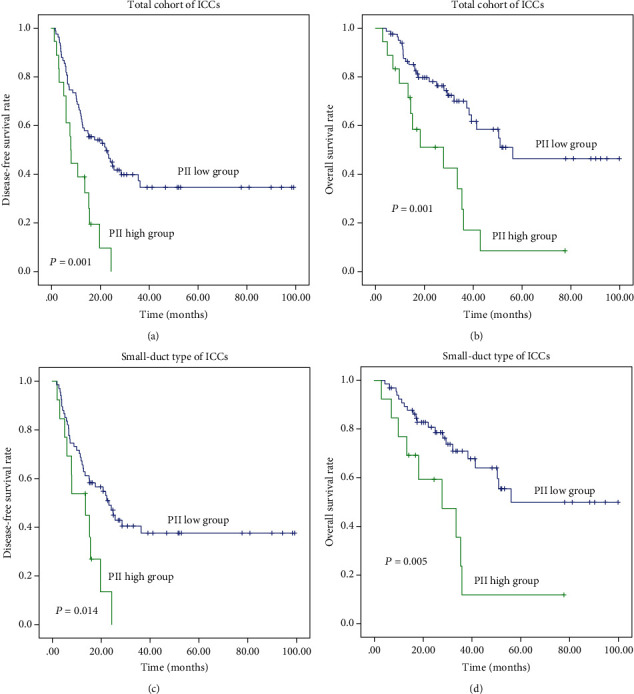
Survival analysis showing patients with high PII had poor prognosis in ICCs (a) and (b) and their small-duct type (c) and (d).

The findings showed that neutrophil, lymphocyte, and monocyte could predict postoperative survival in ICCs. When two of these factors are combined, their predictive ability is better than that of a single factor, such as NLR and LMR. Prognostic inflammatory index (PII) was proposed and defined as neutrophil × monocyte/lymphocyte count (10^9^/L) to fully use the three inflammatory factors that could be easily obtained from routine blood examination. Statistical analysis determined that the cut-off value of PII was 1.50. Univariate analysis showed that ICC cases with high PII had significantly reduced DFS (*P* = 0.001) and OS (*P* = 0.001) compared with those with low PII in ICC (Table 1, Figures 1(a) and 1(b)).

**Figure 2:**
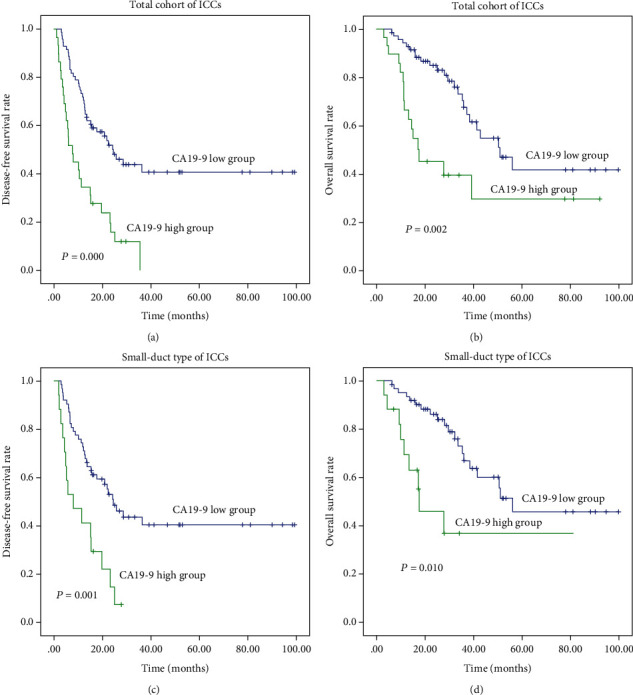
Survival analysis showing patients with high CA19-9 had poor prognosis in ICCs (a) and (b) and their small-duct type (c) and (d).

**Figure 3:**
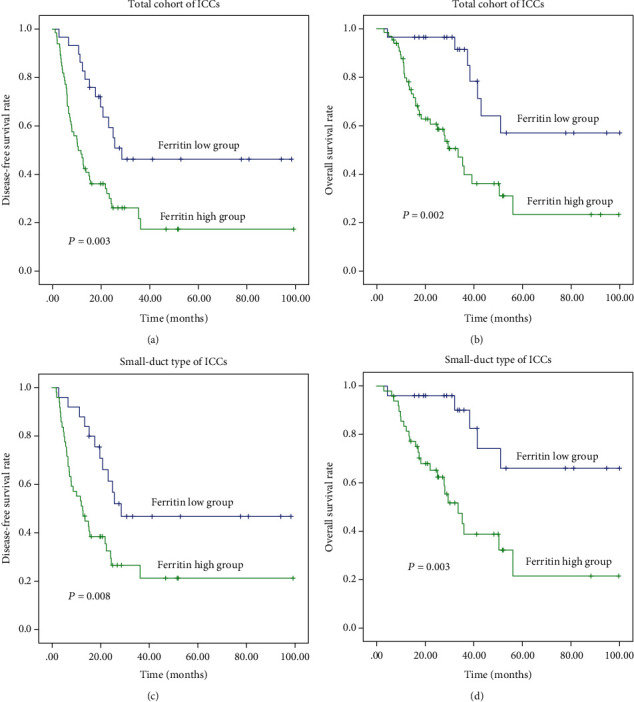
Survival analysis showing patients with high ferritin had poor prognosis in ICCs (a) and (b) and their small-duct type (c) and (d).

In the aspect of tumour marker, survival analysis revealed that increased CEA, CA19-9, CA242, and ferritin were all correlated with reduced OS and DFS in ICC (*P* < 0.025). (Table 1, Figures 2(a), 2(b), 3(a), and 3(b)).

Survival analysis was further conducted for demographic and tumour characteristics. Tumour number, *N* category, *M* category, and TNM stage were all predictors of DFS and OS in the training cohort (*P* < 0.05). Forty-seven patients did not achieve the precise *N* category and TNM stage due to the absence of lymph node dissection. Therefore, tumour number and *M* category were subjected to multivariate analysis (Table S2).

Considering the small sample size (102 cases), a maximum of five variables could be included in the multivariate analysis. Accordingly, CA19-9, tumour number, and *M* category were included in this analysis to study the predictive value of inflammatory factors. The results showed that increased neutrophil and PII were independently associated with inferior DFS (*P* = 0.029, HR (95%CI) = 1.791 (1.063–3.017) and *P* = 0.044, HR (95%CI) = 1.904 (1.017–3.563), respectively) and OS (*P* = 0.025, HR (95%CI) = 2.098 (1.095–4.019) and *P* = 0.001, HR (95%CI) = 3.360 (1.604–7.038), respectively) in the training cohort. In addition, LMR was an independent predictor of OS in patients with ICC (*P* = 0.048, HR (95%CI) = 0.367 (0.136–0.993)) (Table 2).

Meanwhile, to study the predictive value of tumour markers, multivariate analysis included tumour number, *M* category, and PII. The results showed that CEA was an independent predictor of DFS in ICCs (*P* = 0.002, HR (95%CI) = 2.414 (1.383–4.214)). Moreover, the high levels of CA19-9, CA242, and ferritin were all independently associated with reduced DFS (*P* ≤ 0.001, HR (95%CI) = 2.934 (1.696–5.074); *P* = 0.001, HR (95%CI) = 2.737 (1.488–5.036); and *P* = 0.004, HR (95%CI) = 2.534 (1.352–4.750), respectively) and OS (*P* = 0.001, HR (95%CI) = 3.358 (1.668–6.760); *P* = 0.027, HR (95%CI) = 2.348 (1.103–5.001); and *P* = 0.003, HR (95%CI) = 3.779 (1.590–9.077), respectively) in patients with ICC (Table 2).

### 3.2. Prognostic Value of Inflammatory Factors and Tumour Markers for Small-Duct Type ICC in the Training Cohort

In the aspect of inflammatory factors, univariate analysis exhibited that low lymphocyte and high PII were correlated with inferior DFS and OS in small-duct subtype ICC of the training cohort (*P* < 0.045). Furthermore, high NLR and low LMR were risk predictive factors of OS, and high neutrophil was a risk predictor of DFS in small-duct type ICC (*P* < 0.025) (Table 3, Figures 1(c) and 1(d), Figures S2A and B).

In the aspect of tumour marker, univariate analysis revealed that increased CA19-9 and ferritin were correlated with reduced OS and DFS in small-duct type ICC (*P* ≤ 0.10). Moreover, high levels of CEA and CA242 were risk predictive factors of DFS in this type of ICC (*P* ≤ 0.10) (Table 3, Figures 2(c), 2(d), 3(c), and 3(d), and S2C and D).

Univariate analysis was also performed for the prognostic value of the demographic and tumour characteristics in small-duct type ICC. Tumour diameter, tumour number, *T* category, histological grade, vascular invasion, *N* category, *M* category, and TNM stage were all predictors of DFS in small-duct type ICC (*P* < 0.05), whilst only tumour number and *M* category were predictors of DFS and OS (*P* < 0.05). Therefore, the latter two underwent multivariate analysis (Table S3).

The variables included in the multivariate analysis of small-duct type ICC were consistent with those of ICC mentioned above. The results revealed that low level of lymphocyte (*P* = 0.018, HR = 0.450, 95%CI = 0.232–0.870) and high levels of CEA (*P* = 0.010, HR (95%CI) = 2.583 (1.251–5.334)), CA19-9 (*P* = 0.003, HR (95%CI) = 2.946 (1.442–6.020)), and CA242 (*P* = 0.011, HR (95%CI) = 2.988 (1.287–6.938)) were all independently correlated with reduced DFS. Ferritin notably displayed a marginal significance for independently predicting DFS in small-duct type ICC (*P* = 0.054, HR (95%CI) = 2.052 (0.987–4.265)). Meanwhile, low level of LMR (*P* = 0.031, HR (95%CI) = 0.250 (0.071–0.882)) and high levels of PII (*P* = 0.008, HR (95%CI) = 3.215 (1.350–7.655)), CA19-9 (*P* = 0.004, HR (95%CI) = 3.792 (1.542–9.327)), and ferritin (*P* = 0.042, HR (95%CI) = 2.903 (1.037–8.127)) were all independent risk predictors in small-duct type ICC (Table 4).

To sum up, lymphocyte, PII, CA19-9, and ferritin could effectively predict survival outcomes in small-duct type ICC after surgery. Therefore, the association between these factors and the clinical features was analysed. Low lymphocyte was negatively correlated with international normalised ratio of prothrombin time (INR, *P* = 0.026). High PII was correlated with increased INR (*P* = 0.025), alkaline phosphatase (*P* = 0.005), gamma-glutamyltransferase (GGT, *P* = 0.043), frequencies of vascular invasion (*P* = 0.011), and *T* category (*P* = 0.015). High CA19-9 was correlated with decreased albumin (*P* = 0.003) and increased alanine aminotransferase (ALT, *P* = 0.022), total bilirubin (TBIL, *P* = 0.033), *M* category (*P* = 0.044), and TNM stage (*P* = 0.036). High ferritin was correlated with young age (*P* = 0.032), male (*P* = 0.015), and increased INR (*P* = 0.026), ALT (*P* = 0.019), TBIL (*P* = 0.024), GGT (*P* = 0.001), and *T* category (*P* = 0.040) (Tables 5 and S4).

### 3.3. Prognostic Value of Inflammatory Factors and Tumour Markers for Large-Duct Type ICC in the Training Cohort

Survival analysis was also conducted in large-duct type ICC to compare the predictive significance of these factors in small-duct and large-duct subtypes of ICC. Univariate analysis showed that high neutrophil was correlated with decreased OS (*P* = 0.020) and DFS (*P* = 0.048) in large-duct type ICC. Furthermore, high levels of monocyte and PII were risk predictors of DFS (*P* < 0.05). However, all tumour markers were not related to DFS and OS in large-duct type ICC (*P* > 0.05). Multivariate analysis was not performed due to the limited sample size of large-duct type cases (Table S5).

### 3.4. Prognostic Value of Inflammatory Factors and Tumour Markers in Validation Cohort

The validation cohort included 41 males (56.9%) and 31 females (43.1%), and their mean age was 60 years. Among them, 39 cases (38.2%) underwent lymphadenectomy. All demographic and baseline clinicopathological characteristics were balanced between the validation cohort and the training cohort (*P* > 0.05) (Table S1).

The cut-off values were determined, and survival analysis was conducted on inflammatory factors and tumour markers by using the same method. The results were consistent with those produced from the training cohort. Univariate analysis exhibited that neutrophil, NLR, LMR, PII, CEA, and ferritin were all predictors of DFS and OS in patients with ICC (*P* ≤ 0.025). Meanwhile, CA19-9 was a predictive factor of DFS (*P* = 0.028) and associated with a trend of reduced OS (*P* = 0.063) in ICC (Table S6).

Similarly, LMR (*P* ≤ 0.07), PII (*P* ≤ 0.010), and ferritin (*P* ≤ 0.026) were all predictors of DFS and OS in small-duct type ICC. High neutrophil (*P* = 0.018) and CA19-9 (*P* = 0.040) were correlated with shortened DFS in small-duct type ICC (Table S7).

## 4. Discussion

In this study, the predictive value of preoperative inflammatory indices was estimated on the basis of blood routine examination in ICC and its histological subtypes, especially in small-duct type. These factors, including platelet, neutrophil, lymphocyte, monocyte, NLR, LMR, and PLR, are readily available in clinical practice. The above factors were relatively stable in preoperative blood routine examination because these patients had not undergone medical treatment or surgery. However, postoperative blood routine, especially 1 day after the operation, was greatly affected by the factors such as surgical options, operation time, and blood loss, which can also explain that postoperative inflammatory indices cannot be used to predict the survival of ICC patients. In addition, we all know that infection can affect the results of blood routine examination. And cholelithiasis is a risk factor for ICC [6], so ICC patients with cholelithiasis seem likely to cause statistical bias. But this factor should not have influenced the results. There are two acceptable explanations. ICC patients with cholelithiasis were often recognized as large-duct type, and our study focused on the predictive value of inflammatory factors in small duct type. At the same time, such patients are rarely included in this study because the hospital in training cohort is a cancer hospital. Therefore, it is feasible to use preoperative blood sample results to determine the prognosis of small-duct type ICC.

Platelet and PLR were not associated with DFS and OS in small-duct type and the total cohort of ICC, whilst the remaining factors all had a significant predictive value. More importantly, a convenient and efficient inflammatory predictor called PII was proposed to stratify the survival outcome of patients with resectable ICC. Moreover, the prognostic value of tumour markers, such as CA19-9, CEA, CA242, and ferritin, in ICC and its histological subtypes was also evaluated. Only ferritin was negatively correlated with DFS and OS in small-duct type ICC, except for CA19-9, the most commonly used tumour marker for diagnosis and prognosis of ICC.

Inflammatory microenvironment is present before tumour occurs, and the change in inflammatory conditions could promote the development of tumours [25]. Furthermore, systemic and local inflammations could lead to the progression of malignant tumours [26]. Neutrophils could be recruited into ICC tumour through CXCL5 to promote tumour metastasis and recurrence [27]. Many inflammation-based prognostic indices, such as NLR, LMR, GPS, and PI, have been shown to be effective predictors of prognosis in patients with tumours [9–13]. In the present study, the prognostic value of directly available indicators from blood routine examination was assessed in patients with ICC, including platelet, neutrophil, lymphocyte, and monocyte. High neutrophil, low lymphocyte, and high monocyte were all associated with poor prognosis with clear or marginal significance, whilst platelet was not related to postoperative survival in ICC. NLR, LMR, and PLR are the most frequently used inflammation-based prognostic indices in solid tumours; they could be obtained by calculating the ratio of two directly available indicators. Univariate analysis showed that NLR and LMR were predictive factors of DFS and OS in patients with ICC, whereas PLR was not. The platelet-based indicators did not effectively predict postoperative survival, whilst combining two of the other three indicators, such as NLR and LMR, could enhance the predictive power in ICC. Accordingly, PII was proposed for the first time to provide more effective predictive power by using these three indicators simultaneously, as proven in univariate and multivariate analyses. Among the inflammatory markers, only PII was an independent prognostic factor of DFS and OS in ICC.

Previous studies [9, 11, 15, 28] reported that high NLR and low LMR are independent adverse predictors of DFS and OS in patients with ICC and hepatic resection, which was consistent with the results of the present study. However, the predictive value of PLR in ICC remains controversial. Literature [9, 14] from Zhongshan Hospital in China reported that PLR had a negative effect on postoperative survival of ICC. A previous article [15] and the present study found that PLR did not effectively predict survival outcomes in patients with ICC after curative resection. This discrepancy may be due to the different cohorts studied, thus, requiring further study.

The classification of ICC into large-duct and small-duct types has been widely accepted due to its heterogeneity [4]. In the present work, the prognostic value of inflammatory factors in the subtypes of ICC was studied for the first time. Among the directly available indicators, neutrophil was a significant predictor of survival outcomes in the subtypes of ICC, whilst lymphocyte and monocyte were prognostic factors for small-duct and large-duct type ICC, respectively. Similarly, the predictive ability of inflammation-based prognostic indices may be inconsistent between the histological subtypes of ICC. Although NLR and LMR were only correlated with OS in small-duct type ICC, PII was a significant predictor of DFS and OS. High PII was also correlated with poor liver function and advanced tumour stage, which could directly explain the predictive value of PII.

As no tumour-specific markers for ICC have been identified to date, CA19-9 is the most frequently used marker for the diagnosis, prognosis, and detection of recurrence in clinical practice, although it may be affected by biliary obstruction [16–21]. CEA is also a necessary test in postoperative follow-up of patients with ICC [20, 21]. In the present study, CA19-9 and CEA were found to be risk predictors of DFS and OS in ICC, which was consistent with the results of previous reports [17, 18, 21]. However, CEA was not an independent predictor of OS, suggesting that the prognostic value of CEA for DFS was higher than that for OS in patients with ICC. In addition, the two remaining biomarkers (CA242 and ferritin) were independent predictors of DFS and OS, and they could be used to evaluate the prognosis of patients with ICC. To the knowledge of the authors, the effect of ferritin and CA242 on the prognosis of ICC has been rarely reported. Previous studies [29, 30] showed that CA242 could improve the accuracy of ICC diagnosis. Meanwhile, only one literature from the authors' center [24] found that ferritin was an independent predictor of OS in ICC.

Subgroup analysis revealed that CA19-9 and ferritin were independent prognostic factors of DFS and OS, whilst CEA and CA242 were only independently correlated with DFS in small-duct type ICC. Furthermore, high CA19-9 and ferritin were related to poor liver function and advanced tumour stage. Thus, ferritin may be supplementary to CA19-9 in stratifying the survival outcome of patients with small-duct type ICC.

This study has some limitations. First, the sample sizes of the two cohorts were small. Thus, multivariate analysis was not conducted on the predictive value of biomarkers in large-duct type ICC. Second, this study was a retrospective analysis. The predictive value of CA242 was not conformed in the validation cohort.

In conclusion, PII is a convenient and efficient inflammatory predictor of DFS and OS in ICCs and their small-duct type. NLR and LMR, rather than PLR, were correlated with OS in small-duct type ICC. In addition, ferritin may be a supplement to CA19-9 in stratifying survival outcome of patients with small-duct type ICC.

## Figures and Tables

**Table 1:** Univariate analysis of prognostic factors for ICC in the training cohort.

Variables	DFS			OS		
	HR	95% CI	*P* value	HR	95% CI	*P* value
Platelet (10^9/L)	1.450	0.758-2.776	0.262	0.750	0.367-1.535	0.431
(<165 vs. ≥165)						
Neutrophil (10^9/L)	2.183	1.331-3.581	0.002	2.441	1.311-4.544	0.005
(<4.15 vs. ≥4.15)						
Lymphocyte (10^9/L)	0.600	0.368-0.978	0.041	0.564	0.305-1.043	0.068
(<1.60 vs. ≥1.60)						
Monocyte (10^9/L)	1.697	0.970-2.969	0.064	2.307	1.162-4.581	0.017
(<0.54 vs. ≥0.54)						
NLR	2.081	1.156-3.747	0.015	2.698	1.338-5.442	0.006
(<3.00 vs. ≥3.00)						
LMR	0.454	0.215-0.960	0.039	0.275	0.110-0.684	0.006
(<2.70 vs. ≥2.70)						
PLR	1.851	0.987-3.474	0.055	0.910	0.456-1.817	0.789
(<90.0 vs. ≥90.0)						
CEA (ug/L)	2.555	1.518-4.299	≤0.001	2.193	1.120-4.293	0.022
(<4.50 vs. ≥4.50)						
CA19-9 (U/ml)	2.974	1.791-4.936	≤0.001	2.775	1.464-5.262	0.002
(<76.0 vs. ≥76.0)						
CA242 (IU/ml)	2.976	1.688-5.247	≤0.001	2.511	1.235-5.106	0.011
(<30.0 vs. ≥30.0)						
Ferritin (ug/L)	2.495	1.372-4.538	0.003	3.761	1.650-8.572	0.002
(<150.0 vs. ≥150.0)						
PII	2.685	1.498-4.812	0.001	3.161	1.628-6.138	0.001
(<1.50 vs. ≥1.50)						

**Table 2:** Multivariate analysis of prognostic factors for ICC in the training cohort.

Variables	DFS			OS		
	HR	95% CI	*P* value	HR	95% CI	*P* value
Inflammatory factors						
Neutrophil (10^9/L)	1.791	1.063-3.017	0.029	2.098	1.095-4.019	0.025
(<4.15 vs. ≥4.15)						
Lymphocyte (10^9/L)	0.594	0.332-1.061	0.078			
(<1.60 vs. ≥1.60)						
Monocyte (10^9/L)				2.042	0.884-4.714	0.095
(<0.54 vs. ≥0.54)						
NLR	1.554	0.801-3.013	0.192	2.117	0.944-4.748	0.069
(<3.00 vs. ≥3.00)						
LMR	0.772	0.339-1.758	0.537	0.367	0.136-0.993	0.048
(<2.70 vs. ≥2.70)						
PII	1.904	1.017-3.563	0.044	3.360	1.604-7.038	0.001
(<1.50 vs. ≥1.50)						
Tumour markers						
CEA (ug/L)	2.414	1.383-4.214	0.002	1.851	0.856-4.001	0.118
(<4.50 vs. ≥4.50)						
CA19-9 (U/ml)	2.934	1.696-5.074	≤0.001	3.358	1.668-6.760	0.001
(<76.0 vs. ≥76.0)						
CA242 (IU/ml)	2.737	1.488-5.036	0.001	2.348	1.103-5.001	0.027
(<30.0 vs. ≥30.0)						
Ferritin (ug/L)	2.534	1.352-4.750	0.004	3.779	1.590-9.077	0.003
(<150.0 vs. ≥150.0)						

**Table 3:** Univariate analysis of prognostic factors for small-duct type ICC in the training cohort.

Variables	DFS			OS		
	HR	95% CI	*P* value	HR	95% CI	*P* value
Platelet (10^9/L)	1.185	0.574-2.447	0.646	0.646	0.287-1.455	0.292
(<165 vs. ≥165)						
Neutrophil (10^9/L)	2.033	1.143-3.576	0.015	1.976	0.954-4.090	0.067
(<4.15 vs. ≥4.15)						
Lymphocyte (10^9/L)	0.495	0.281-0.872	0.015	0.476	0.231-0.982	0.044
(<1.60 vs. ≥1.60)						
Monocyte (10^9/L)	1.061	4.495-2.273	0.878	1.496	0.567-3.949	0.416
(<0.54 vs. ≥0.54)						
NLR	1.877	0.949-3.714	0.071	2.642	1.161-6.019	0.021
(<3.00 vs. ≥3.00)						
LMR	0.394	0.152-1.021	0.055	0.218	0.070	0.008
(<2.70 vs. ≥2.70)						
PLR	1.791	0.889-3.608	0.103	0.862	0.395-1.883	0.710
(<90.0 vs. ≥90.0)						
PII	2.367	1.186-4.724	0.014	3.127	1.420-6.886	0.005
(<1.50 vs. ≥1.50)						
CEA (ug/L)	2.383	1.229-4.619	0.010	1.731	0.698-4.292	0.236
(<4.50 vs. ≥4.50)						
CA19-9 (U/ml)	2.956	1.586-5.508	0.001	2.858	1.287-6.347	0.010
(<76.0 vs. ≥76.0)						
CA242 (IU/ml)	3.254	1.544-6.859	0.002	2.073	0.779-5.516	0.145
(<30.0 vs. ≥30.0)						
Ferritin (ug/L)	2.466	1.270	0.008	4.391	1.658-11.629	0.003
(<150.0 vs. ≥150.0)						

**Table 4:** Multivariate analysis of prognostic factors for small-duct type ICC in the training cohort.

Variables	DFS			OS		
	HR	95% CI	*P* value	HR	95% CI	*P* value
Inflammatory factors						
Neutrophil (10^9/L)	1.498	0.801-2.801	0.206			
(<4.15 vs. ≥4.15)						
Lymphocyte (10^9/L)	0.450	0.232-0.870	0.018	0.495	0.217-1.131	0.095
(<1.60 vs. ≥1.60)						
NLR				1.666	0.543-5.110	0.372
(<3.00 vs. ≥3.00)						
LMR				0.250	0.071-0.882	0.031
(<2.70 vs. ≥2.70)						
PII	1.674	0.792-3.540	0.177	3.215	1.350-7.655	0.008
(<1.50 vs. ≥1.50)						
Tumour markers						
CEA (ug/L)	2.583	1.251-5.334	0.010			
(<4.50 vs. ≥4.50)						
CA19-9 (U/ml)	2.946	1.442-6.020	0.003	3.792	1.542-9.327	0.004
(<76.0 vs. ≥76.0)						
CA242 (IU/ml)	2.988	1.287-6.938	0.011			
(<30.0 vs. ≥30.0)						
Ferritin (ug/L)	2.052	0.987-4.265	0.054	2.903	1.037-8.127	0.042
(<150.0 vs. ≥150.0)						

**Table 5:** Association of PII and ferritin with demographic and clinicopathological characteristics in small-duct type ICC of training cohort.

Variables	PII			Ferritin		
	Low	High	*P* value	Low	High	*P* value
Age (years)			0.289			0.032
<65	52 (81.3%)	12 (18.8%)		16 (27.6%)	42 (72.4%)	
≥65	15 (93.8%)	1 (6.3%)		9 (56.3%)	7 (43.8%)	
Gender			0.711			0.015
Male	39 (84.8%)	7 (15.2%)		10 (22.7%)	34 (77.3%)	
Female	28 (82.4%)	6 (17.6%)		15 (50.0%)	15 (50.0%)	
INR			0.025			0.026
<1.0	43 (91.5%)	4 (8.5%)		19 (44.2%)	24 (55.8%)	
≥1.0	24 (72.7%)	9 (27.3%)		6 (19.4%)	25 (80.6%)	
ALT (U/L)			0.069			0.019
<40	54 (88.5%)	7 (11.5%)		23 (41.1%)	33 (58.9%)	
≥40	13 (68.4%)	6 (31.6%)		2 (11.1%)	16 (88.9%)	
Albumin (g/L)			0.115			0.279
<43	20 (74.1%)	7 (25.9%)		7 (25.9%)	20 (74.1%)	
≥43	47 (88.7%)	6 (11.3%)		18 (38.3%)	29 (61.7%)	
TBIL (umol/L)			0.052			0.024
<21	61 (87.1%)	9 (12.9%)		25 (38.5%)	40 (61.5%)	
≥21	6 (60.0%)	4 (40.0%)		0 (0.0%)	9 (100.0%)	
GGT (U/L)			0.043			0.001
<45	36 (92.3%)	3 (7.7%)		19 (52.8%)	17 (47.2%)	
≥45	31 (75.6%)	10 (24.4%)		6 (15.8%)	32 (84.2%)	
ALP (U/L)			0.005			0.197
<135	60 (89.6%)	7 (10.4%)		19 (52.8%)	17 (47.2%)	
≥135	7 (53.8%)	6 (46.2%)		6 (15.8%)	32 (84.2%)	
Histological grade			0.328			0.254
G1-G2	36 (80.0%)	9 (20.0%)		16 (40.0%)	24 (60.0%)	
G3	30 (88.2%)	4 (11.8%)		9 (27.3%)	24 (72.7%)	
Vascular invasion			0.011			0.293
Negative	40 (93.0%)	3 (7.0%)		16 (40.0%)	24 (60.0%)	
Positive	25 (71.4%)	10 (28.6%)		9 (28.1%)	23 (71.9%)	
Satellite lesions			1.000			0.411
Negative	59 (83.1%)	12 (16.9%)		24 (35.8%)	43 (64.2%)	
Positive	8 (88.9%)	1 (11.1%)		1 (14.3%)	6 (85.7%)	
*T* category			0.015			0.040
*T*1	35 (94.6%)	2 (5.4%)		16 (45.7%)	19 (54.3%)	
*T*2 − *T*4	32 (74.4%)	11 (25.6%)		9 (23.1%)	30 (76.9%)	
*N* category			1.000			0.119
*N*0	23 (76.7%)	7 (23.3%)		12 (42.9%)	16 (57.1%)	
*N*1	7 (77.8%)	2 (22.2%)		1 (11.1%)	8 (88.9%)	
*M* category			0.417			0.320
*M*0	65 (84.4%)	12 (15.6%)		25 (35.2%)	46 (64.8%)	
*M*1	2 (66.7%)	1 (33.3%)		0 (0.0%)	3 (100.0%)	
TNM stage			0.669			0.065
I-II	23 (79.3%)	6 (20.7%)		12 (44.4%)	15 (55.6%)	
III-IV	7 (70.0%)	3 (30.0%)		1 (10.0%)	9 (90.0%)	

## Data Availability

The datasets used and/or analysed during this study are available from the corresponding author on reasonable request.

## Supplementary Materials

Supplementary Materials Figure S1: Kaplan-Meier curves showing patients with high neutrophil had reduced DFS (a) and OS (b) in the total cohort of ICCs. Figure S2: Kaplan-Meier curves showing patients with low lymphocyte (a), high CEA (c), and high CA242 (d) had reduced DFS, whilst low LMR (b) was associated with decreased OS in small-duct type. Table S1: comparison of demographic and clinicopathological characteristics between the training cohort and the validation cohort. Table S2: univariate analysis of demographic and clinicopathological factors for ICC in the training cohort. Table S3: univariate analysis of demographic and clinicopathological factors for small-duct type ICC in the training cohort. Table S4: association of lymphocyte and CA19-9 with demographic and clinicopathological characteristics in small-duct type ICC of training cohort. Table S5: univariate analysis of prognostic factors for large-duct type ICC in the training cohort. Table S6: the cutoff values and univariate analysis of prognostic factors for ICC in the validation cohort. Table S7: univariate analysis of prognostic factors for small-duct type ICC in the validation cohort.
