# Supplementary material for: Prognostic Value of Inflammatory and Tumour Markers in Small-Duct Subtype Intrahepatic Cholangiocarcinoma after Curative-Intent Resection
Source: Gastroenterol Res Pract. 2021 Mar 26;2021:6616062. doi: 10.1155/2021/6616062 (PMC8018878; doi:10.1155/2021/6616062)
Supplement: Supplementary Materials — Figure S1: Kaplan-Meier curves showing patients with high neutrophil had reduced DFS (a) and OS (b) in the total cohort of ICCs. Figure S2: Kaplan-Meier curves showing patients with low lymphocyte (a), high CEA (c), and high CA242 (d) had reduced DFS, whilst low LMR (b) was associated with decreased OS in small-duct type. Table S1: comparison of demographic and clinicopathological characteristics between the training cohort and the validation cohort. Table S2: univariate analysis of demographic and clinicopathological factors for ICC in the training cohort. Table S3: univariate analysis of demographic and clinicopathological factors for small-duct type ICC in the training cohort. Table S4: association of lymphocyte and CA19-9 with demographic and clinicopathological characteristics in small-duct type ICC of training cohort. Table S5: univariate analysis of prognostic factors for large-duct type ICC in the training cohort. Table S6: the cutoff values and univariate analysis of prognostic factors for ICC in the validation cohort. Table S7: univariate analysis of prognostic factors for small-duct type ICC in the validation cohort. [file 6616062.f1.docx]

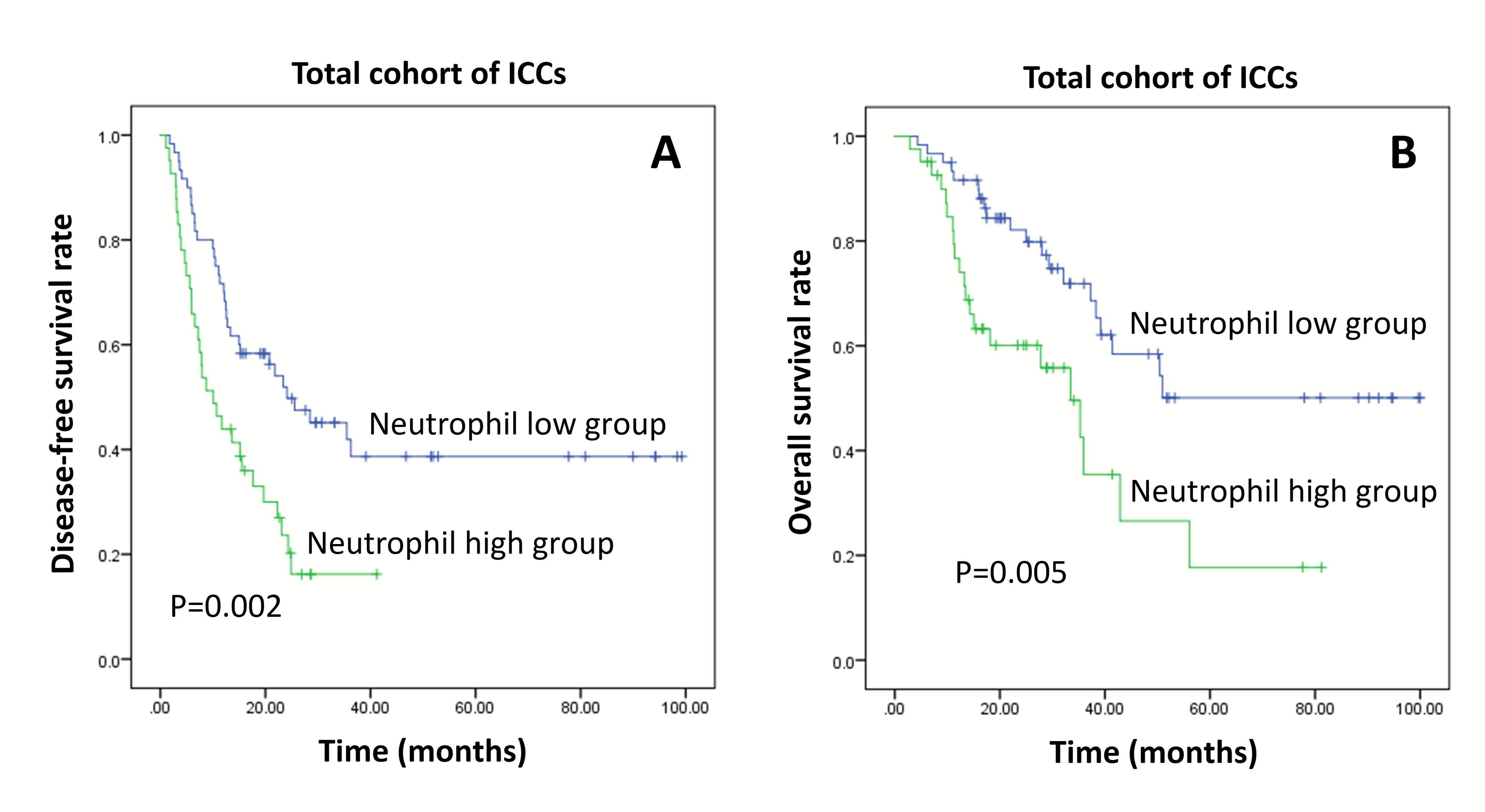


**Figure S1.** Kaplan-Meier curves showing patients with high neutrophil had reduced DFS (A) and OS (B) in total cohort of ICCs.


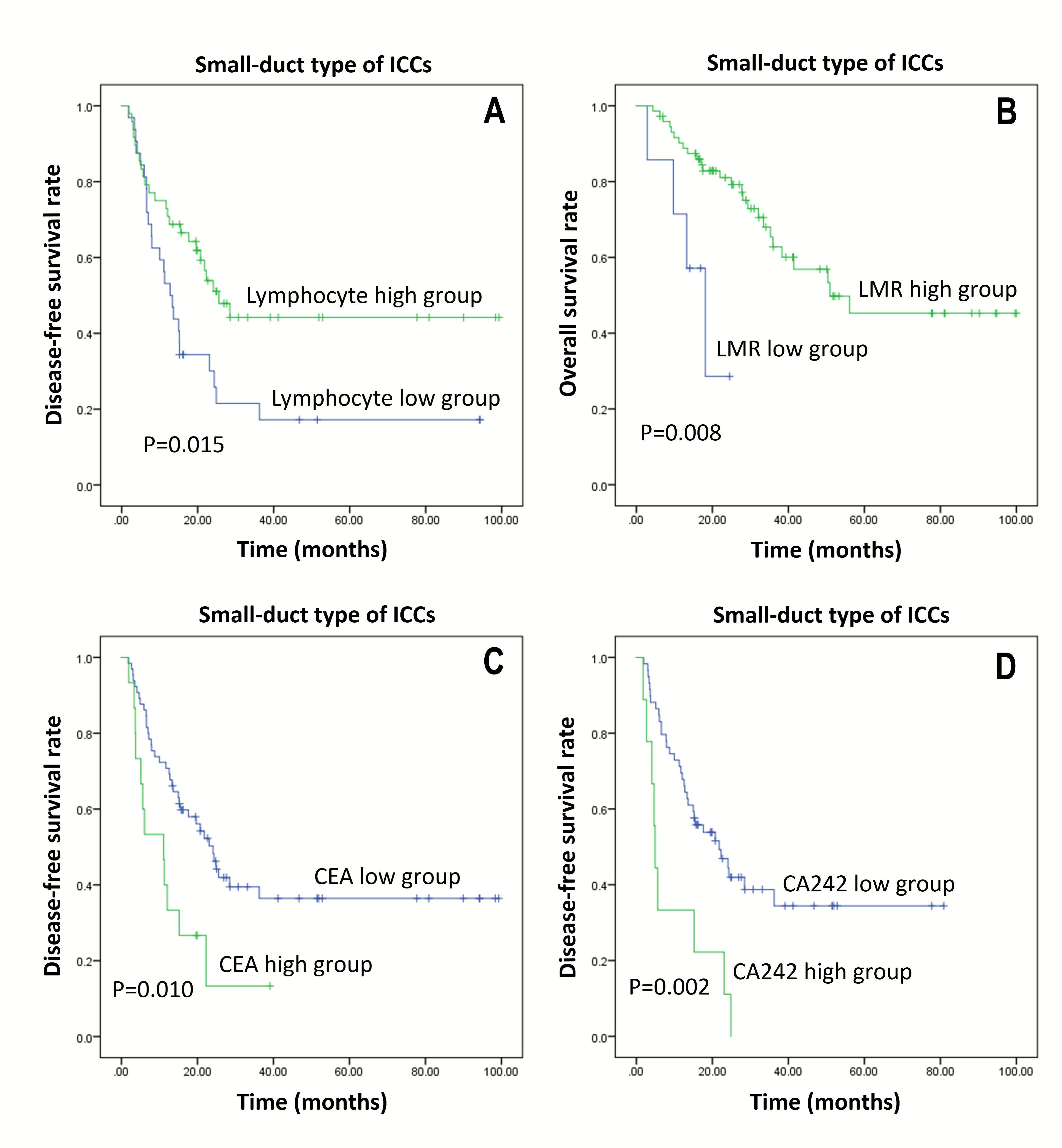


**Figure S2.** Kaplan-Meier curves showing patients with low lymphocyte (A), high CEA (C), and high CA242 (D) had reduced DFS, while low LMR (B) was associated with decreased OS in small-duct type of ICCs.

**Table S1** Comparison of demographic and clinicopathological characteristics between the training cohort and the validation cohort

| **Variables** | **Training cohort** (n=102) | **Validation cohort** (n=72) | **P value** |
| --- | --- | --- | --- |
| **Age** (years) | 49(28-77) | 59(32-76) | 0.937 |
| **Gender** (Male/Female) | 57/45 | 41/31 | 0.889 |
| **Platelet** (10^9/L) | 226(66-428) | 228(83-441) | 0.920 |
| **Neutrophil** (10^9/L) | 3.88(1.19-12.34) | 3.60(1.10-7.30) | 0.540 |
| **Lymphocyte** (10^9/L) | 2.80(0.77-3.57) | 1.62(0.82-3.14) | 0.505 |
| **Monocyte** (10^9/L) | 0.43(0.18-0.85) | 0.44(0.21-0.80) | 0.995 |
| **CEA** (ug/L) | 2.32(0.28-46.36) | 2.42(0.29-23.85) | 0.591 |
| **CA19-9** (U/ml) | 26.73(0.60-56509.00) | 32.07(0.69-1200.0) | 0.366 |
| **CA242** (IU/ml) | 8.43(0.98-708.28) | 9.17(1.34-700.0) | 0.408 |
| **Ferritin** (ug/L) | 184.5(13.8-1263.0) | 185.1(13.5-1126.0) | 0.881 |
| **INR** | 0.97(0.79-1.17) | 0.97(0.77-1.20) | 0.902 |
| **Albumin** (g/L) | 44.2(31.5-54.9) | 43.9(32.5-54.0) | 0.558 |
| **ALT** (U/L) | 24(6-674) | 23(5-645) | 0.886 |
| **TBIL** (umol/L) | 14.1(6.8-287.0) | 13.2(6.4-276) | 0.503 |
| **GGT** (U/L) | 56(13-1263) | 60(15-1136) | 0.617 |
| **ALP** (U/L) | 94(39-997) | 99(42-978) | 0.609 |
| **Histological Grade** (G1-G2/G3) | 59/42 | 37/35 | 0.359 |
| **T Category** (T1/T2-T4) | 44/58 | 25/47 | 0.264 |
| **N Category** (N0/N1) | 37/18 | 27/15 | 0.758 |
| **M Category** (M0/M1) | 94/8 | 66/6 | 0.907 |

**Table S2** Univariate analysis of demographic and clinicopathological factors for ICC in the training cohort

| **Variables** | **DFS** | | |  | **OS** | | |
| --- | --- | --- | --- | --- | --- | --- | --- |
|  | HR | 95%CI | P Value |  | HR | 95%CI | P Value |
| **Age** (year) | 1.011 | 0.566-1.803 | 0.972 |  | 1.484 | 0.756-2.916 | 0.252 |
| (＜65 vs. ≥65) |  |  |  |  |  |  |  |
| **Gender** | 0.869 | 0.534-1.415 | 0.572 |  | 0.743 | 0.402-1.374 | 0.344 |
| (Male vs. Female) |  |  |  |  |  |  |  |
| **Tumor** **diameter** (cm) | 1.708 | 1.033-2.824 | **0.037** |  | 1.494 | 0.789-2.827 | 0.218 |
| (＜4.5 vs. ≥4.5) |  |  |  |  |  |  |  |
| **Tumor number** | 2.596 | 1.369-4.925 | **0.003** |  | 2.360 | 1.117-4.982 | **0.024** |
| (Single vs. Multiple) |  |  |  |  |  |  |  |
| **Histological Grade** | 1.792 | 1.092-2.939 | **0.021** |  | 1.604 | 0.861-2.988 | 0.137 |
| (G1-G2 vs. G3) |  |  |  |  |  |  |  |
| **Vascular invasion** | 1.487 | 0.909-2.433 | 0.114 |  | 1.274 | 0.682-2.378 | 0.447 |
| (Negative vs. Postive) |  |  |  |  |  |  |  |
| **Nerve invasion** | 1.181 | 0.653-2.138 | 0.582 |  | 1.029 | 0.474-2.234 | 0.943 |
| (Negative vs. Postive) |  |  |  |  |  |  |  |
| **Satellite lesions** | 0.582 | 0.320-1.055 | 0.075 |  | 0.969 | 0.446-2.107 | 0.937 |
| (Negative vs. Postive) |  |  |  |  |  |  |  |
| **T Category** | 1.944 | 1.158-3.261 | **0.012** |  | 1.844 | 0.939-3.619 | 0.075 |
| (T1 vs. T2-T4) |  |  |  |  |  |  |  |
| **N Category** | 3.362 | 1.722-6.563 | **≤0.001** |  | 2.473 | 1.099-5.565 | **0.029** |
| (N0 vs. N1) |  |  |  |  |  |  |  |
| **M Category** | 4.328 | 1.978-9.474 | **≤0.001** |  | 3.031 | 1.263-7.279 | **0.013** |
| (M0 vs. M1) |  |  |  |  |  |  |  |
| **TNM stage** | 4.386 | 2.228-8.634 | **≤0.001** |  | 3.858 | 1.694-8.785 | **0.001** |
| (I-II vs. III-IV) |  |  |  |  |  |  |  |

**Table S3** Univariate analysis of demographic and clinicopathological factors for small-duct type ICC in the training cohort

| **Variables** | **DFS** | | |  | **OS** | | |
| --- | --- | --- | --- | --- | --- | --- | --- |
|  | HR | 95%CI | P Value |  | HR | 95%CI | P Value |
| **Age** (year) | 0.757 | 0.367-1.565 | 0.453 |  | 1.138 | 0.487-2.661 | 0.766 |
| (＜65 vs. ≥65) |  |  |  |  |  |  |  |
| **Gender** | 0.904 | 0.516-1.586 | 0.726 |  | 0.839 | 0.408-1.722 | 0.631 |
| (Male vs. Female) |  |  |  |  |  |  |  |
| **Tumor** **diameter** (cm) | 1.900 | 1.040-3.471 | **0.037** |  | 2.135 | 0.949-4.803 | 0.067 |
| (＜4.5 vs. ≥4.5) |  |  |  |  |  |  |  |
| **Tumor number** | 3.863 | 1.817-8.213 | **≤0.001** |  | 3.455 | 1.461-8.173 | **0.005** |
| (Single vs. Multiple) |  |  |  |  |  |  |  |
| **Histological Grade** | 2.008 | 1.129-3.572 | **0.018** |  | 1.899 | 0.909-3.971 | 0.088 |
| (G1-G2 vs. G3) |  |  |  |  |  |  |  |
| **Vascular invasion** | 1.853 | 1.049-3.275 | **0.034** |  | 1.473 | 0.706-3.072 | 0.302 |
| (Negative vs. Postive) |  |  |  |  |  |  |  |
| **Nerve invasion** | 0.973 | 0.455-2.081 | 0.945 |  | 0.506 | 0.153-1.675 | 0.264 |
| (Negative vs. Postive) |  |  |  |  |  |  |  |
| **Satellite lesions** | 0.494 | 0.219-1.117 | 0.090 |  | 0.852 | 0.295-2.464 | 0.768 |
| (Negative vs. Postive) |  |  |  |  |  |  |  |
| **T Category** | 2.452 | 1.350-4.453 | **0.003** |  | 2.179 | 0.995-4.774 | 0.052 |
| (T1 vs. T2-T4) |  |  |  |  |  |  |  |
| **N Category** | 2.422 | 1.028-5.707 | **0.043** |  | 2.169 | 0.737-6.384 | 0.160 |
| (N0 vs. N1) |  |  |  |  |  |  |  |
| **M Category** | 5.344 | 1.556-18.353 | **0.008** |  | 8.340 | 1.778-39.129 | **0.007** |
| (M0 vs. M1) |  |  |  |  |  |  |  |
| **TNM stage** | 2.704 | 1.168-6.258 | **0.020** |  | 2.809 | 0.991-7.960 | 0.052 |
| (I-II vs. III-IV) |  |  |  |  |  |  |  |

**Table S4** Association of lymphocyte and CA19-9 with demographic and clinicopathological characteristics in small-duct type ICC of training cohort

| **Variables** | **Lymphocyte** | | |  |  | **CA19-9** | | |
| --- | --- | --- | --- | --- | --- | --- | --- | --- |
|  | Low | High | P Value |  |  | Low | High | P Value |
| **Age** (years) |  |  | 0.052 |  |  |  |  | 0.704 |
| ＜65 | 29(45.3%) | 35(54.7%) |  |  |  | 50(79.4%) | 13(20.6%) |  |
| ≥65 | 3(18.8%) | 13(81.3%) |  |  |  | 12(75.0%) | 4(25.0%) |  |
| **Gender** |  |  | 0.268 |  |  |  |  | 0.618 |
| Male | 16(34.8%) | 30(52.9%) |  |  |  | 37(80.4%) | 9(19.6%) |  |
| Female | 16(47.1%) | 18(52.9%) |  |  |  | 25(75.8%) | 8(24.2%) |  |
| **INR** |  |  | **0.026** |  |  |  |  | 0.238 |
| ＜1.0 | 14(29.8%) | 33(70.2%) |  |  |  | 39(83.0%) | 8(17.0%) |  |
| ≥1.0 | 18(54.5%) | 15(45.5%) |  |  |  | 23(71.9%) | 9(28.1%) |  |
| **Albumin** (g/L) |  |  | 0.288 |  |  |  |  | **0.003** |
| ＜43 | 13(48.1%) | 14(51.9%) |  |  |  | 16(59.3%) | 11(40.7%) |  |
| ≥43 | 19(35.8%) | 34(64.2%) |  |  |  | 46(88.5%) | 6(11.5%) |  |
| **ALT** (U/L) |  |  | 0.830 |  |  |  |  | **0.022** |
| ＜40 | 24(39.3%) | 37(60.7%) |  |  |  | 51(85.0%) | 9(15.0%) |  |
| ≥40 | 8(42.1%) | 11(57.9%) |  |  |  | 11(57.9%) | 8(42.1%) |  |
| **TBIL** (umol/L) |  |  | 0.732 |  |  |  |  | **0.033** |
| ＜21 | 27(38.6%) | 43(61.4%) |  |  |  | 57(82.6%) | 12(17.4%) |  |
| ≥21 | 5(50.0%) | 5(50.0%) |  |  |  | 5(50.0%) | 5(50.0%) |  |
| **GGT** (U/L) |  |  | 0.784 |  |  |  |  | 0.063 |
| ＜45 | 15(38.5%) | 24(61.5%) |  |  |  | 34(87.2%) | 5(12.8%) |  |
| ≥45 | 17(41.5%) | 24(58.5%) |  |  |  | 28(70.0%) | 12(30.0%) |  |
| **ALP** (U/L) |  |  | 0.083 |  |  |  |  | 0.139 |
| ＜135 | 24(35.8%) | 43(64.2%) |  |  |  | 54(81.8%) | 12(18.2%) |  |
| ≥135 | 8(61.5%) | 5(38.5%) |  |  |  | 8(61.5%) | 5(38.5%) |  |
| **Histological Grade** |  |  | 0.440 |  |  |  |  | 0.744 |
| G1-G2 | 16(35.6%) | 29(64.4%) |  |  |  | 35(79.5%) | 9(20.5%) |  |
| G3 | 15(44.1%) | 19(55.9%) |  |  |  | 26(76.5%) | 8(23.5%) |  |
| **Vascular invasion** |  |  | 0.612 |  |  |  |  | 0.409 |
| Negative | 16(37.2%) | 27(62.8%) |  |  |  | 35(81.4%) | 8(18.6%) |  |
| Postive | 15(42.9%) | 20(57.1%) |  |  |  | 25(73.5%) | 9(26.5%) |  |
| **Satellite lesions** |  |  | 0.773 |  |  |  |  | 0.190 |
| Negative | 28(39.4%) | 43(60.6%) |  |  |  | 54(76.1%) | 17(23.9%) |  |
| Postive | 4(44.4%) | 5(55.6%) |  |  |  | 8(100.0%) | 0(0.0%) |  |
| **T Category** |  |  | 0.927 |  |  |  |  | 0.983 |
| T1 | 15(40.5%) | 22(59.5%) |  |  |  | 29(78.4%) | 8(21.6%) |  |
| T2-T4 | 17(39.5%) | 26(60.5%) |  |  |  | 33(78.6%) | 9(21.4%) |  |
| **N Category** |  |  | 1.000 |  |  |  |  | 0.170 |
| N0 | 12(40.0%) | 18(60.0%) |  |  |  | 24(80.0%) | 6(20.0%) |  |
| N1 | 4(44.4%)) | 5(55.6%) |  |  |  | 4(50.0%) | 4(50.0%) |  |
| **M Category** |  |  | 1.000 |  |  |  |  | **0.044** |
| M0 | 31(40.3%) | 46(59.7%) |  |  |  | 62(80.5%) | 15(19.5%) |  |
| M1 | 1(33.3%) | 2(66.7%) |  |  |  | 0(0.0%) | 2(100.0%) |  |
| **TNM stage** |  |  | 0.711 |  |  |  |  | **0.036** |
| I-II | 11(37.9%) | 18(62.1%) |  |  |  | 24(82.8%) | 5(17.2%) |  |
| III-IV | 5(50.0%) | 5(50.0%) |  |  |  | 4(44.4%) | 5(55.6%) |  |

**Table S5** Univariate analysis of prognostic factors for large-duct type ICC in the training cohort

| **Variables** | **DFS** | | |  | **OS** | | |
| --- | --- | --- | --- | --- | --- | --- | --- |
|  | HR | 95%CI | P Value |  | HR | 95%CI | P Value |
| **Platelet** (10^9/L) | 2.560 | 0.576-11.380 | 0.217 |  | 1.125 | 0.226-5.598 | 0.885 |
| (＜165 vs. ≥165) |  |  |  |  |  |  |  |
| **Neutrophil** (10^9/L) | 3.464 | 1.211-9.904 | **0.020** |  | 3.535 | 1.010 | **0.048** |
| (＜4.15 vs. ≥4.15) |  |  |  |  |  |  |  |
| **Lymphocyte** (10^9/L) | 1.435 | 0.517-3.981 | 0.448 |  | 0.828 | 0.238-2.878 | 0.766 |
| (＜1.60 vs. ≥1.60) |  |  |  |  |  |  |  |
| **Monocyte** (10^9/L) | 4.265 | 1.446-12.578 | **0.009** |  | 2.807 | 0.807-9.762 | 0.105 |
| (＜0.54 vs. ≥0.54) |  |  |  |  |  |  |  |
| **NLR** | 3.238 | 0.987-10.624 | 0.053 |  | 2.929 | 0.719-11.935 | 0.134 |
| (＜3.00 vs. ≥3.00) |  |  |  |  |  |  |  |
| **LMR** | 0.571 | 0.160-2.038 | 0.382 |  | 0.469 | 0.094-2.348 | 0.357 |
| (＜2.70 vs. ≥2.70) |  |  |  |  |  |  |  |
| **PLR** | 1.629 | 0.367-7.234 | 0.521 |  | 0.772 | 0.155-3.840 | 0.752 |
| (＜90.0 vs. ≥90.0) |  |  |  |  |  |  |  |
| **PII** | 4.047 | 1.251-13.092 | **0.020** |  | 1.989 | 0.558-7.094 | 0.289 |
| (＜1.50 vs. ≥1.50) |  |  |  |  |  |  |  |
| **CEA** (ug/L) | 2.231 | 0.789-6.412 | 0.130 |  | 1.943 | 0.559-6.756 | 0.296 |
| (＜4.50 vs. ≥4.50) |  |  |  |  |  |  |  |
| **CA19-9** (U/ml) | 2.613 | 0.880-7.756 | 0.084 |  | 1.788 | 0.517-6.184 | 0.359 |
| (＜76.0 vs. ≥76.0) |  |  |  |  |  |  |  |
| **CA242** (IU/ml) | 2.696 | 0.856-8.488 | 0.090 |  | 2.918 | 0.721-11.818 | 0.133 |
| (＜30.0 vs. ≥30.0) |  |  |  |  |  |  |  |
| **Ferritin** (ug/L) | 2.414 | 0.539-10.803 | 0.249 |  | 2.169 | 0.456-10.306 | 0.330 |
| (＜150.0 vs. ≥150.0) |  |  |  |  |  |  |  |

**Table S6** The cutoff values and univariate analysis of prognostic factors for ICC in the validation cohort

| **Variables** | cut-off value | **DFS** | | |  | **OS** | | |
| --- | --- | --- | --- | --- | --- | --- | --- | --- |
|  |  | HR | 95%CI | P Value |  | HR | 95%CI | P Value |
| **Platelet** (10^9/L) | 180 | 1.135 | 0.605-2.129 | 0.694 |  | 0.782 | 0.371-1.648 | 0.517 |
| **Neutrophil** (10^9/L) | 4.15 | 2.817 | 1.595-4.976 | **≤0.001** |  | 2.603 | 1.308-5.182 | **0.006** |
| **Lymphocyte** (10^9/L) | 1.70 | 0.707 | 0.407-1.227 | 0.218 |  | 0.815 | 0.410-1.619 | 0.559 |
| **Monocyte** (10^9/L) | 0.39 | 2.049 | 1.122-3.741 | **0.020** |  | 1.655 | 0.805-3.406 | 0.171 |
| **NLR** | 3.00 | 2.210 | 1.124-4.343 | **0.021** |  | 2.580 | 1.156-5.755 | **0.021** |
| **LMR** | 2.65 | 0.368 | 0.155-0.874 | **0.023** |  | 0.210 | 0.077-0.569 | **0.002** |
| **PLR** | 90.0 | 1.387 | 0.675-2.850 | 0.373 |  | 0.773 | 0.347-1.719 | 0.527 |
| **PII** | 1.60 | 3.435 | 1.708-6.907 | **0.001** |  | 3.119 | 1.446-6.725 | **0.004** |
| **CEA** (ug/L) | 4.30 | 2.397 | 1.336-4.301 | **0.003** |  | 2.310 | 1.112-4.797 | **0.025** |
| **CA19-9** (U/ml) | 43.0 | 1.848 | 1.068-3.197 | **0.028** |  | 1.920 | 0.966-3.815 | 0.063 |
| **Ferritin** (ug/L) | 140.0 | 2.787 | 1.352-5.744 | **0.005** |  | 6.146 | 1.849-20.428 | **0.003** |

**Table S7** Univariate analysis of prognostic factors for small-duct type ICC in the validation cohort

| **Variables** | **DFS** | | |  | **OS** | | |
| --- | --- | --- | --- | --- | --- | --- | --- |
|  | HR | 95%CI | P Value |  | HR | 95%CI | P Value |
| **Platelet** (10^9/L) | 0.891 | 0.439-1.811 | 0.750 |  | 0.641 | 0.267-1.536 | 0.319 |
| (＜180 vs. ≥180) |  |  |  |  |  |  |  |
| **Neutrophil** (10^9/L) | 2.250 | 1.150-4.401 | **0.018** |  | 1.871 | 0.803-4.359 | 0.146 |
| (＜4.15 vs. ≥4.15) |  |  |  |  |  |  |  |
| **Lymphocyte** (10^9/L) | 0.513 | 0.263-1.000 | 0.050 |  | 0.685 | 0.292-1.606 | 0.384 |
| (＜1.70 vs. ≥1.70) |  |  |  |  |  |  |  |
| **Monocyte** (10^9/L) | 1.991 | 1.000-3.964 | 0.050 |  | 1.750 | 0.736-4.161 | 0.205 |
| (＜0.39 vs. ≥0.39) |  |  |  |  |  |  |  |
| **NLR** | 1.812 | 0.788-4.169 | 0.162 |  | 2.386 | 0.875-6.510 | 0.089 |
| (＜3.00 vs. ≥3.00) |  |  |  |  |  |  |  |
| **LMR** | 0.167 | 0.046-0.611 | **0.007** |  | 0.104 | 0.027-0.401 | **0.001** |
| (＜2.65 vs. ≥2.65) |  |  |  |  |  |  |  |
| **PLR** | 1.418 | 0.621-3.237 | 0.407 |  | 0.748 | 0.292-1.917 | 0.545 |
| (＜90.0 vs. ≥90.0) |  |  |  |  |  |  |  |
| **PII** | 3.230 | 1.364-7.646 | **0.008** |  | 3.472 | 1.353-8.909 | **0.010** |
| (＜1.60 vs. ≥1.60) |  |  |  |  |  |  |  |
| **CEA** (ug/L) | 1.630 | 0.734-3.620 | 0.230 |  | 1.601 | 0.577-4.448 | 0.366 |
| (＜4.30 vs. ≥4.30) |  |  |  |  |  |  |  |
| **CA19-9** (U/ml) | 1.999 | 1.034-3.865 | **0.040** |  | 1.943 | 0.827-4.561 | 0.127 |
| (＜43.0 vs. ≥43.0) |  |  |  |  |  |  |  |
| **Ferritin** (ug/L) | 2.445 | 1.111-5.382 | **0.026** |  | 7.747 | 1.758-34.137 | **0.007** |
| (＜140.0 vs. ≥140.0) |  |  |  |  |  |  |  |
